# Genetic Analysis and Predictive Modeling of COVID-19 Severity in a Hospital-Based Patient Cohort

**DOI:** 10.3390/biom15030393

**Published:** 2025-03-10

**Authors:** Iraide Alloza-Moral, Ane Aldekoa-Etxabe, Raquel Tulloch-Navarro, Ainhoa Fiat-Arriola, Carmen Mar, Eloisa Urrechaga, Cristina Ponga, Isabel Artiga-Folch, Naiara Garcia-Bediaga, Patricia Aspichueta, Cesar Martin, Aitor Zarandona-Garai, Silvia Pérez-Fernández, Eunate Arana-Arri, Juan-Carlos Triviño, Ane Uranga, Pedro-Pablo España, Koen Vandenbroeck-van-Caeckenbergh

**Affiliations:** 1 Inflammation & Biomarkers Group, Biobizkaia Health Research Institute, 48903 Barakaldo, Spain; iraide.alloza@ehu.eus (I.A.-M.); ane.aldekoaetxabe@bio-bizkaia.eus (A.A.-E.); raquel.tulloch@gmail.com (R.T.-N.); ainhoa.fiatarriola@bio-bizkaia.eus (A.F.-A.); cesar.martin@ehu.eus (C.M.); 2 Physiology Department, Faculty of Medicine and Nursery, Basque Country University (UPV/EHU), 48940 Leioa, Spain; patricia.aspichueta@ehu.eus; 3 Red de Enfermedades Inflamatorias (REI), Redes de Investigación Cooperativa Orientada a Resultados en Salud (RICORS), Carlos IIII Health Research Institute, 28029 Madrid, Spain; 4 Pneumology Department, Galdakao-Usansolo University Hospital, Biobizkaia Health Research Institute, 48960 Galdakao, Spain; mariadelcarmen.marmedina@osakidetza.eus (C.M.); eloisamaria.urrechagaigartua@osakidetza.eus (E.U.); cristina.pongapalacio@osakidetza.eus (C.P.); ane.urangaecheverria@osakidetza.eus (A.U.); espanapedropablo@gmail.com (P.-P.E.); 5 Bioinformatic Unit, Biobizkaia Health Research Institute, 48903 Barakaldo, Spain; naiarabediaga@gmail.com (N.G.-B.); aitor.zarandonagarai@bio-bizkaia.eus (A.Z.-G.); silvia.perezfernandez@bio-bizkaia.eus (S.P.-F.); 6 Research Center for the Study of Liver and Gastrointestinal Diseases (CIBERehd), 28029 Madrid, Spain; 7 Biobizkaia Health Research Institute, Cruces University Hospital, 48903 Barakaldo, Spain; 8 Biochemistry and Molecular Biology Department, Science and Technology School, Basque Country University (UPV/EHU), 48940 Leioa, Spain; 9 Biofisika Institute (UPV/EHU, CSIC), 48940 Leioa, Spain; 10 Clinical Epidemiology Unit, Biobizkaia Health Research Institute, Cruces University Hospital, Plaza de Cruces s/n, 48903 Barakaldo, Spain; eunate.aranaarri@osakidetza.eus; 11 Bioinformatics Department, Sistemas Genómicos, 46980 Peterna, Spain; jc.trivino@sistemasgenomicos.com; 12 Ikerbasque, Basque Foundation for Science, 48013 Bilbao, Spain

**Keywords:** SARS-CoV-2, GWAS, COVID-19, severity, HLA, KIF19

## Abstract

The COVID-19 pandemic has had a devastating impact, with more than 7 million deaths worldwide. Advanced age and comorbidities partially explain severe cases of the disease, but genetic factors also play a significant role. Genome-wide association studies (GWASs) have been instrumental in identifying loci associated with SARS-CoV-2 infection. Here, we report the results from a >820 K variant GWAS in a COVID-19 patient cohort from the hospitals associated with IIS Biobizkaia. We compared intensive care unit (ICU)-hospitalized patients with non-ICU-hospitalized patients. The GWAS was complemented with an integrated phenotype and genetic modeling analysis using HLA genotypes, a previously identified COVID-19 polygenic risk score (PRS) and clinical data. We identified four variants associated with COVID-19 severity with genome-wide significance (rs58027632 in KIF19; rs736962 in HTRA1; rs77927946 in DMBT1; and rs115020813 in LINC01283). In addition, we designed a multivariate predictive model including HLA, PRS and clinical data which displayed an area under the curve (AUC) value of 0.79. Our results combining human genetic information with clinical data may help to improve risk assessment for the development of a severe outcome of COVID-19.

## 1. Introduction

The global impact of the severe acute respiratory syndrome coronavirus 2 (SARS-CoV-2) pandemic has been devastating, with over 7.07 million reported deaths to date (https://covid19.who.int/, accessed on 5 December 2024). The COVID-19 pandemic caused by this virus started with a series of patients with pneumonia of unknown cause in December 2019 in Wuhan, Hubei province, China [1,2]. Nowadays, there is a consensus that COVID-19 affects the body systemically and that the disease displays a wide range of clinical presentations. These span from asymptomatic cases, which make up about one-third of infections, to severe illness that can ultimately result in fatality [3,4]. While advanced age, male sex, and comorbidities such as hypertension and diabetes are established risk factors [5], these factors alone do not explain the diverse manifestations of the disease. Interactions between SARS-CoV-2 and bacteria via co-infection or modulation of microbiota may contribute to the disease [6,7]. Uncovering the genetic determinants of the host response to SARS-CoV-2 infection may offer a promising path towards a more complete understanding of the disease [8]. Until today, genome-wide association studies (GWASs) have uncovered over 50 genetic regions linked to susceptibility, hospitalization and severity in relation to COVID-19 [9]. Among the candidate regions identified by GWAS, the 3p21.31 locus (SLC6A20, LZTFL1, CCR9, FYCO1, CXCR6, XCR1, CCR1, CCR2, CCR5 and CCR3) has shown strong association with increased risk to develop severe COVID-19 [10]. This section of the chromosome was reported as being inherited from H. neanderthalensis to modern humans [11,12].

GWASs have been instrumental in studies of complex diseases for the identification of novel risk loci and biological pathways for biomarker identification. However, since most complex diseases are highly polygenic, each of the SNPs identified in a GWAS usually have a small effect on the phenotype of interest [13]. Polygenic risk score (PRS) methods have been developed to estimate individual genetic risk for a specific condition based on combined SNPs. These PRS can be implemented in clinical prediction or screening [14]. Recently, Horowitz and colleagues have designed a PRS to identify those individuals who have a higher risk to develop a severe form of COVID-19 by analyzing 756.000 individuals from four cohorts [15]. The PRS included 12 variants and was associated with a 1.58-fold higher risk.

Characterizing genetic variants associated with COVID-19 severity will not only shed light on the biological mechanisms underlying the disease but could also help with identifying predictive/prognostic biomarkers as well as facilitating the development of more effective prevention and treatment strategies. Here, we elaborate on both a GWAS analysis and an integrated phenotype and genetic analysis (PRS and HLA) based on genotyped data obtained with the Axiom^TM^ Human Genotyping SARS-CoV-2 Array in a hospital-based COVID-19 patient cohort.

## 2. Materials and Methods

### 2.1. Patient Cohort

The patients included in this study were recruited from Biobizkaia Research Health Institute-associated hospitals (Galdakao-Usansolo University Hospital and Cruces University Hospital) in the course of 2020 and were all residents of Biscay province in northern Spanish Basque Country. All enrolled participants (n = 1176) were hospitalized individuals diagnosed specifically with the SARS-CoV-2 disease. Nasopharyngeal swab samples were processed with RT-PCR kits, SARS-CoV-2 Cobas 6800 Roche kit (Roche Diagnostics, Rotkreuz, Switzerland) or GeneXpert Kit (Cepheid, Sunnyvale, CA, USA) to test for positivity of SARS-CoV-2. The severity of the disease was defined based on admission to the intensive care unit (ICU), i.e., patients presenting with respiratory failure and/or hemodynamic instability and/or multiple organ failure (involving two or more organs). All patients were informed about the details of this study, and only patients who had given consent were included. This study was approved by the local ethics committee (CEIm-E, code PI-CES-BIOEF-2020-08). All participants consented to participate in this study in compliance with the Helsinki declaration.

### 2.2. Genomic DNA Preparation and Genotyping

Genomic DNA (gDNA) samples from patients, hospitalized with COVID-19, were obtained from the Biobanco Vasco. gDNA was extracted from 200 μL of blood using QIAamp^®^ DNA Blood Mini Kit (Qiagen, Hilden, Germany). gDNA purity was analyzed by absorbance using the NanoDrop^TM^ One spectrophotometer (ThermoFisher Scientific, Waltham, MA, USA). gDNA concentration was quantified using Qubit™ dsDNA HS (High Sensitivity) Assay Kit (ThermoFisher Scientific, USA). Only samples with gDNA OD260/OD280 ratio of 1.8–2.0 and OD260/OD230 ratio > 1.5 were included in the analysis. gDNA was adjusted to a final concentration of 10 ng/µL. Potential degradation of DNA samples was assessed by 1% agarose gel electrophoresis before array analysis, and degraded samples were not further processed.

DNA aliquots were sent to the Spanish Genotyping National Center (CeGen) for genotyping with the GenTitan platform (ThermoFisher Scientific, USA) using the Axiom^TM^ Human Genotyping SARS-CoV-2 Research Array (ThermoFisher Scientific, USA), which includes >820,000 variants. The array includes markers from pathways associated with immunology, inflammation, respiratory distress and cardiovascular disease, variants of cell surface receptors, virus entry facilitators and interactors and pharmacogenomic markers.

### 2.3. Genotyping Data Quality Control

Raw data were processed with the Axiom Analysis Suite software (version 5.3.0.45, ThermoFisher Scientific, UK) following import of the cell files generated by the GenTitan platform (ThermoFisher Scientific, USA). Genotype calling was performed using the Best Practice Workflow, which carries out quality control (QC) analysis for samples and plates and only performs genotype calling on samples that pass the determined QC thresholds. QC was performed using PLINK (version 1.90 beta) [16], with criteria including the removal of SNPs if call rate < 95% and dish QC < 98%, inbreeding coefficient > 0.2 or if sex discrepancy was identified. Furthermore, based on initial genotype data, the variants which were disproportionately missing between cases and controls (*p*-value < 10^−5^), exhibited a minor allele frequency (MAF) < 1%, or showed extensive deviation from Hardy–Weinberg equilibrium (*p*-value < 10^−6^ for controls, *p*-value < 10^−10^ for cases), were excluded. Individuals deviating more than 3 times the standard deviation from the heterozygosity rate mean were also excluded. The dataset was analyzed for cryptic relatedness, calculating identity by descent (IBD) based on the LD-pruned SNPs on the autosomal chromosomes. A value of 0.2 was used as the threshold. Individuals with greater call rate between/for each pair with PI_HAT value > 0.2 were excluded. We analyzed our dataset using 1000 Genomes Data as a guide for population stratification. Principal component analysis (PCA) values in both LD-pruned datasets were calculated. Samples matching European ancestry (Figure S1) were kept for GWAS. The final quality-controlled dataset was composed of 929 samples (132 cases and 797 controls) and 513,379 variants. Whole-genome imputation was conducted with BEAGLE v5.4 [17] for chromosomes 1-22 and X. To impute chromosome X, we coded males as diploid in the non-pseudoautosomal regions (non-PAR regions).

### 2.4. Genome-Wide Association Analysis

Single-trait (ICU vs. non-ICU) GWAS analysis was carried out using the statgenGWAS package (version 1.0.9) [18,19], a linear mixed-model genome-wide test for association, with adjustments to control for potential population stratification. Gender and diabetes mellitus were used as covariants. Manhattan and QQ plots were generated with the statgenGWAS package’s plot function. Ideogram was generated with Richie Lab Visualization application (https://visualization.ritchielab.org/phenograms/plot, accessed on 5 December 2024). Functional analysis was performed on variants associated with COVID-19 severity (*p*-value < 10^−4^) by means of the Multi-marker Analysis of GenoMic Annotation (MAGMA) [20].

### 2.5. Analysis of the COVID-19 3p21.31 Locus

Based on previous reports [10,11], we examined the Axiom^TM^ Human Genotyping SARS-CoV-2 Research Array genotype data corresponding to 12 genes located at the 3p21.31 locus. Association analysis was carried out for each individual gene (ICU-hospitalized vs. non-ICU-hospitalized patients). Codominant, dominant, recessive, overdominant and additive models were evaluated. The analysis was performed using the SNPassoc library (version 2.1-0) in R (4.3.3).

### 2.6. Analysis of HLA Allele Association

Four-digit HLA types were imputed from the SARS-CoV-2 genotype array using the Axiom™ HLA Analysis software (ThermoFisher Scientific, UK), and HLA-A, -B, -C, -DPA1, -DPB1, -DQA1, -DQB1 and -DRB1/3/4/5 alleles of each locus were assigned to samples. A threshold of 0.7 was set up to the posterior probability to create a marker representing the presence or absence of each four-digit HLA allele for each individual [21]. We compared the HLA allele frequencies between the ICU-hospitalized patients with COVID-19 and non-ICU-hospitalized patients with COVID-19. The HLA alleles were collapsed using the first two levels, and then the case–control association was performed using sex and age as covariables. The significance of HLA alleles was evaluated for precision and significance equilibrium adjusted by means of the FDR method.

### 2.7. SNP Identification for PRS Replication Analysis

We assessed the PRS previously reported by Horowitz et al. [15] associated with severity of COVID-19 disease. Since the 12 SNPs used in this PRS were absent from the Axiom^TM^ Human Genotyping SARs-CoV-2 Research Array, we searched for alternative proxies to be used in our replication analysis. Linkage disequilibrium values of all proxies for the 12 candidate SNPs were obtained with the Ldlink tool (https://ldlink.nih.gov, accessed on 5 December 2024) based on the Caucasian population (CEU) described in the 1000 Genomes project [22]. Proxies with r^2^ > 0.6 included in our array were identified using the LdlinkR library (version 1.4.0) in R (version 4.3.3). Table S1 includes the identities of the original 12 SNPs, their proxies present in the array and corresponding r^2^ LD values, as well as the individual ORs and weights in the PRS.

### 2.8. Polygenic Risk Scoring and Model Estimation: Univariable and Multivariable Method

The PRS estimator was calculated following the strategy described by Horowitz et al. [15]. Raw PRS values for each sample were normalized using the median of controls, and variance was adjusted to 1. The OR for each individual risk factor was calculated using logistic regression. The discriminative capacity of these factors was evaluated by calculating the area under the receiver operating characteristic curve (AUC) using the pROC method [23]. For the construction of the multivariable model, a forward stepwise regression was applied for selection of the best risk factors [24]. The OR of each risk factor in the multivariable model was calculated using a regression model adjusted by two principal components.

## 3. Results

### 3.1. Identification and Exploration of GWAS Variants in a COVID-19 Hospital-Based Patient Cohort

**Figure 1:**
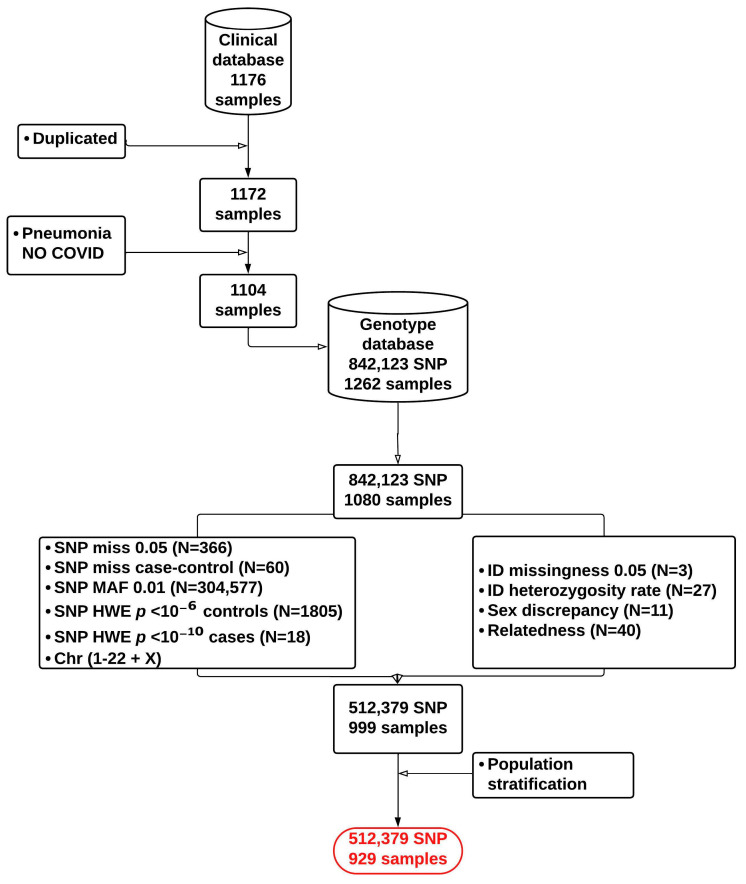
Flow chart showing quality control steps implemented in the GWAS of COVID-19 severity.

**Figure 2:**
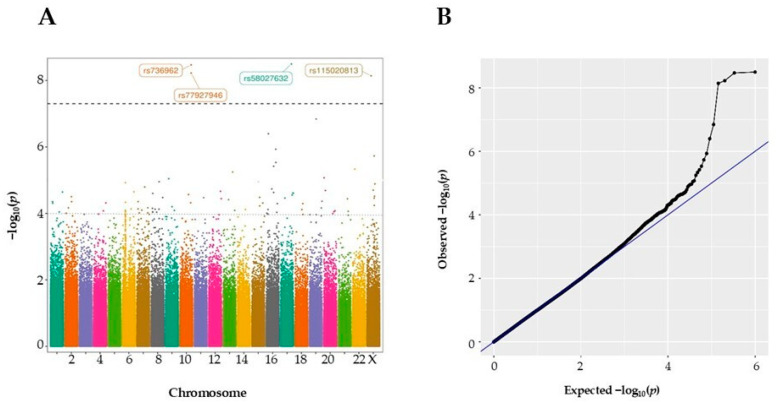
GWAS results for comparison of variant allele frequencies in ICU- and non-ICU-based hospitalized COVID-19 patients. (**A**) Manhattan plot for severity of COVID-19 disease. The horizontal black dotted line is drawn at GWAS significance threshold level (*p* < 5 × 10^−8^), and the grey dotted line corresponds to the suggestive threshold of *p* < 10 ^−4^. Each dot represents a single SNP, and the contrasting colors of each block show the extent of each chromosome. (**B**) Q–Q plot of GWAS results. Expected *p*-value is represented with a blue line and observed *p*-value with a black line.

The demographic and clinical details of the individuals enrolled in this study are shown in Table 1. Genotyping analysis was carried out using the Axiom™ Human Genotyping SARS-CoV-2 Research Array. GWAS analysis for COVID-19 severity was performed by analyzing variant allele distribution in ICU-hospitalized vs. non-ICU-hospitalized patients. After performing QC, GWAS analysis included 929 patient samples and 512,379 variants (Figure 1). After imputation adjusted for gender and diabetes, and taking into account the results from the principal component analysis (PCA), comparison of ICU and non-ICU variant allele frequencies revealed four variants showing genome-wide association (*p*-value < 5 × 10^−8^) (Figure S1 and Figure 2).

In Figure 2A, a Manhattan plot of the identified GWAS variants is presented, with the chromosomal position of the variants on the *x*-axis, and *p*-values on the *y*-axis shown in the −log_10_ scale. The Q–Q plot shows that the *p*-values obtained deviated significantly from the expected results, indicating that the identified effects go beyond random variation and may have significant association with COVID-19 severity (Figure 2B). The four variants identified with genome-wide significance are located in loci 17q25.1 (rs58027632, *p*-value 3.1 × 10^−9^, OR = 1.26, KIF19); 10q26 (rs736962, *p*-value 3.04 × 10^−9^, OR = 1.49, HTRA1; rs77927946, *p*-value 5.98 × 10^−9^, OR = 1.50, DMBT1); and Xp11.4 (rs115020813, *p*-value 7.23 × 10^−9^, OR = 1.34, LINC01283) (Table 2).

**Figure 3:**
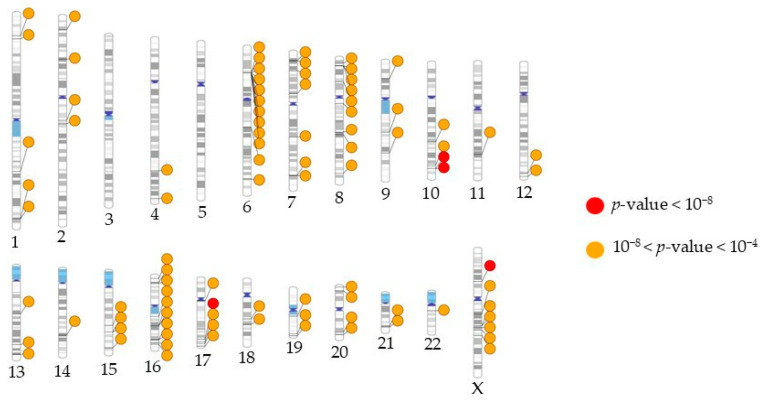
Chromosome ideogram representing regions in the genome with the COVID-19-severity-associated variants identified by GWAS in this study. Lines are plotted on the chromosomes corresponding to the genomic location of each SNP, connecting to colored circles representing their significance values, *p*-value ≤ 10^−8^ (red), or *p*-value between 10^−8^ and 10^−4^ (orange). The bands in gradations of grey in each chromosome represent heterochromatin. The dark blue regions are the areas around the centromere, and the light blue areas represent variable regions.

**Figure 4:**
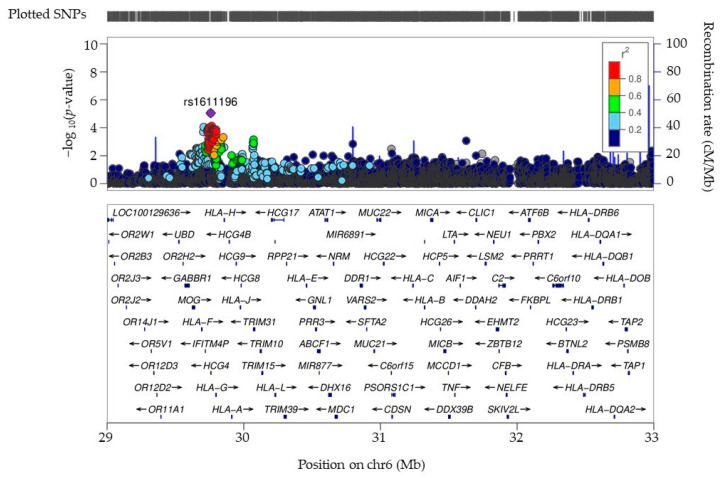
Regional association plot of the HLA region on chromosome 6. The index SNP (purple diamond) represents the lead HLA variant identified in the GWAS for COVID-19 severity. Surrounding SNPs are color-coded based on their degree of linkage disequilibrium (LD) with the index SNP, using r^2^ values derived from the 1000 Genomes Project (European population). The *x*-axis displays genomic coordinates (hg19), and the *y*-axis represents the −log10 (*p*-value) of association for each SNP. Gene annotations are provided below the plot. Arrows indicate transcriptional direction, and squares reflect length and structure of the corresponding genes.

Eighty-seven additional variants were associated with severity with suggestive levels of significance (10^−8^ < *p* < 10^−4^). These include variants at previously reported loci such as 16q23.1 (WWOX [25]) and 6p22.1 (HLA-G [26]) (full variant information in Table S2). The ideogram image in Figure 3 shows the chromosomal locations of all suggestive and genome-wide variants. Among the suggestive variants, we observed nine loci with at least two associated markers (Table 3). LINC01139, CYP1A2 and CPLX3 have been identified in the BioGRID COVID-19 Coronavirus Curation Project (https://thebiogrid.org, accessed on 30 November 2024). CYP1A2 variants have also been previously identified as associated with severity in COVID-19 disease [27]. The HLA-G gene contained 10 associated variants, all of which were in LD (lead variant rs1611196). These variants are also in LD with rs1610696, which was reported, similarly to our results, to have a protective association with COVID-19 severity (Table S2) [26]. A regional association plot of the HLA region, showing a peak of association at the HLA-G locus, is presented in Figure 4. Of the 72 distinct loci marked by the SNPs from our GWAS with *p* < 10^−4^, twenty-one have been associated with COVID-19 previously (Table S2). MAGMA pathway analysis revealed galactosyltransferase activity as significantly associated with COVID-19 severity (beta = 0.67; *p* = 2 × 10^−4^). This activity has been reported to facilitate the assembly and secretion of the SARS-CoV-2 virus within the host [28]. Additional relevant findings of this analysis highlight the pancreas (beta = 0.017; *p*-value = 0.03) and pituitary gland (beta = 0.014; *p* = 0.07), both of which have been related with COVID-19 severity before [29,30].

The 3p21.31 locus has been identified as a critical genetic region associated with severe COVID-19 outcomes, particularly affecting the risk of hospitalization and the development of severe symptoms such as respiratory failure [10]. This locus includes several genes that play roles in immune response, chemokine signaling and other critical biological processes relevant to viral pathogenesis. In our GWAS, this region, and chromosome 3 at large, did not yield any variants with genome-wide or suggestive significance (Figure 3). To further explore potential genotype effects, we examined the Axiom™ Human Genotyping SARS-CoV-2 Research Array genotype data corresponding to this chromosomal region. Genetic variants associated with the 3p21.31 locus were considered, including those linked to genes such as CCR1, CCR3, CCR9, CXCR6, FYCO1, LARS2, LIMD1, LZTFL1, SACM1L, SLC6A20, XCR1 and CCR5. The codominant model analysis identified nine SNPs borderline significantly associated (*p* < 0.05) with COVID-19 severity, involving six of the twelve studied genes (Table S3).

### 3.2. Association of HLA Alleles with COVID-19 Severity

The relationship between human leukocyte antigen (HLA) alleles and COVID-19 severity has been a topic of extensive research, with evidence suggesting that certain HLA alleles may influence the clinical outcomes of those infected with SARS-CoV-2 [31]. To further elucidate this potential connection, we examined HLA allele distribution from the Axiom^TM^ Human Genotyping SARS-CoV-2 Research Array genotype data using Axiom™ HLA Analysis software. This analysis revealed significant associations between specific HLA alleles and COVID-19 severity (Table 4). Certain alleles were linked to a heightened risk of severe disease in our cohort, while others appeared to confer protection against severe outcomes. For instance, the HLA allele DRB1*13:03 exhibited a significantly increased risk of severe COVID-19, with an odds ratio (OR) of 3.765 (*p*-value = 0.043). Similarly, DQB1*06:09 (OR = 3.604, *p*-value = 0.045) and B*45:01 (OR = 3.422, *p*-value = 0.031) were also associated with a higher risk of severe disease. Conversely, the HLA allele DQB1*05:02 was associated with a reduced risk of severe COVID-19 (OR = 0.128, *p*-value = 0.043), as were A*01:01 (OR = 0.431, *p*-value = 0.002) and C*02:02 (OR = 0.432, *p*-value = 0.036).

### 3.3. Comprehensive Model Combining PRS, HLA and Phenotype for Predicting Severe COVID-19 Risk

The possible significance between every variable and phenotype was analyzed using the conditional and non-conditional *p*-value obtained from logistic regression, odds ratio and AUC. The SNPs in our array identified as proxies of those from the Horowitz PRS [15] with an LD value of r^2^ > 0.6 for the Caucasian population are listed in Table S1. The PRS obtained with these proxies was normalized and classified as a dichotomous variable based on the 0.9 quartile. We calculated the PCAs to correct for population bias in the cohort, and the first 10 were selected for a statistical relationship with the phenotype using the Kruskal–Wallis test. PC2 and PC3 were the only significant PCs (*p* < 0.05) and were incorporated into the multivariable model as independent variables.

**Figure 5:**
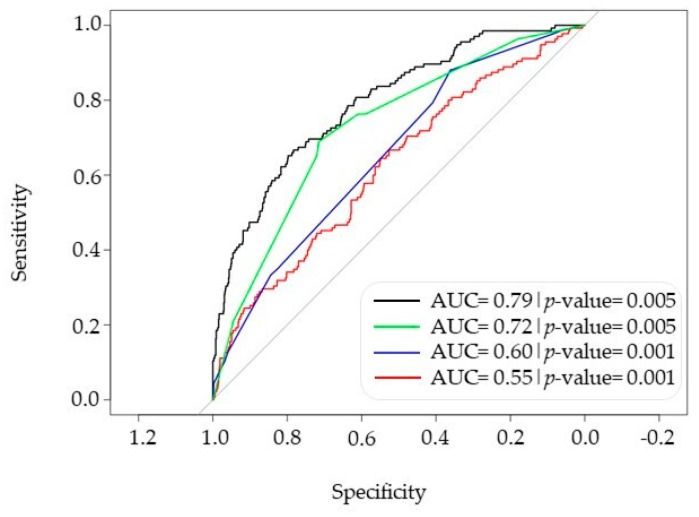
ROC curves for COVID-19 severity. Model 1, PRS + HLA + phenotype (black line); model 2, PRS (red line); model 3, HLA genotypes (blue line); model 4, phenotypes (green line). The grey line is the baseline for non-association. ROC, receiver operating characteristic.

The results of the univariable analysis for PRS and phenotype are shown in Table S4. PRS analysis alone yielded a *p*-value of 0.001. Gender, diabetes and oxygen therapy also were found to be statistically significant in this univariate analysis. We performed multivariable analysis using PRS, phenotype and HLA genotypes to identify the group of individuals at higher risk to develop severe COVID-19. In this case, the HLA risk alleles (OR > 1) were grouped into one estimator, named HLA_pos, and protective alleles (OR < 1) into another estimator, named HLA_neg (Table S5). Following the best-fit models protocol reported before [32], we obtained a model which, according to Hosmer and Lemeshow’s goodness-of-fit statistic test [33], was stable without evidence of overfitting. The receiver operating characteristic (ROC) curve for this model yielded an AUC of 0.793, indicating that the discriminative power of the global multivariable model designed here is stronger than that of the PRS, phenotypes or HLA genetics model individually (Figure 5). Additionally, we conducted a PRS analysis incorporating the proxies of Horowitz’s SNPs along with our GWAS top variants. The results showed no improvement in the AUC compared to using Horowitz’s PRS proxies alone [AUC (PRS Horowitz’s proxies) = 0.55 vs. AUC (PRS Horowitz’s proxies + our top GWAS variants) = 0.55]. These results could be due to the small size of our cohort and possible bias from it. Corroboration of these SNPs in independent and bigger cohorts would allow, in the future, for their possible real impact on improved PRS development to be assessed. Nevertheless, as shown above, the incorporation of HLA information into Horowitz’s PRS enhanced the predictive performance of the model without introducing collinearity.

## 4. Discussion

Throughout the COVID-19 pandemic, it was observed that individuals who were initially considered at low risk (young and without comorbidities) could develop severe illness, while those deemed high risk sometimes experienced only mild symptoms or even remained asymptomatic without requiring hospitalization or additional medical care [34,35]. The underlying cause of these contrasting outcomes is partly attributed to specific sequence variations in individual genomes [36]. The varying manifestations of COVID-19 are primarily determined by a complex interplay between host genetic variants and non-genetic factors, such as age, sex, body mass index and socioeconomic characteristics [37].

In this study, we identified by a GWAS approach four variants associated with genome-wide significance with severe COVID-19 by comparing ICU with non-ICU hospital-based patients. All patients included in this study were identified as SARS-CoV-2-positive by RT-PCR, and all were hospitalized strictly as a consequence of COVID-19 complications. Rs58027632 (OR = 1.26, *p*-value = 3.19 × 10^−9^) is an intronic variant located in the KIF19 gene, which is involved in regulation of the length of motile cilia. Cilia are located on the surface of respiratory epithelial cells, forming the first contact point between the host and, in this case, the SARS-CoV-2 virus. Previous investigations have demonstrated that coronaviruses are capable of modifying the expression of genes related to respiratory cilia, causing aberrant cilia structure (i.e., length) that may lead to respiratory diseases [38]. Chen et al. 2023 [39] identified KIF19 as one of the biomarkers included in logistic regression models to assess COVID-19 severity. Their models use KIF19 in pairs with other biomarkers to evaluate its relation to disease progression and its impact on prediction accuracy. Although additional research studying the functional role of the SNPs in KIF19 is needed, this SNP may contribute to the severity of COVID-19 by impairing correct cilia function, which is essential for removing the virus from the respiratory tract. Also, our GWAS identified one intergenic variant located on the X chromosome (Xp11.4 (rs115020813, *p*-value 7.23 × 10^−9^, OR = 1.34, LINC01283)). This variant lies next to a gene (BCOR), which codifies for a protein involved in Th17 cell formation [40]. Finally, the other two identified GWAS variants were located on locus 10q26 (rs736962, *p*-value 3.04 × 10^−9^, OR = 1.49, HTRA1; rs77927946, *p*-value 5.98 × 10^−9^, OR = 1.50; DMBT1). The proteins transcribed by the genes in which these two variants are located (i.e., HTRA1 and DMBT1) are SARS-CoV-2-binding proteins [41,42]. The GWAS catalog database shows that several genomic variants in HTRA1 have been previously associated with COVID-19 [25,43]. Rs736962 and rs77927946 from our GWAS, and those reportedly associated with COVID-19 in the GWAS catalog, are in LD (D′ > 0.7).

Locus 3p21.31, which has been identified by previous GWAS analysis as significantly linked to severe COVID-19 disease [44,45], did not emerge from our GWAS. The specific set-up of our study (which did not consider healthier, i.e., not hospitalized or asymptomatic, COVID-19 patients) and the limited sample size could underlie the lack of replication of this and various other previously reported risk loci. Further validation in independent cohorts will be useful to confirm the findings from the present study.

Individuals with COVID-19 show dysregulated immune responses, evident in hyperinflammation and a cytokine storm [46]. These processes are thought to mediate the immunopathogenesis of COVID-19 and the associated morbidity and mortality [47]. HLA alleles, located on the short arm of chromosome 6, are crucial immune mediators of viral infection [48] and may play a role in modifying the response to SARS-CoV-2 infection. We here show that, in our cohort, specific HLA alleles appeared to be associated with higher or lower risk of developing severe COVID-19. In particular, allele A*01:01 showed in our cohort an OR of 0.43, similar to that found previously [49]. Moreover, risk allele DRB3*03.01 has previously been shown to be associated with severity [50].

Finally, we tested the possibility of using a multivariate model containing genetic information (PRS and HLA) and clinical information to identify patients at higher risk of developing a severe form of the disease. We considered three types of risk factors in the modeling effort, i.e., PRS, HLA and phenotypic risk factors such as the use of ventimasks and glasses. All these factors showed statistical significance in our cohort, although with relatively low odds ratios and discriminant capacity. This discriminant capacity makes it possible to assess the practical application of these individual risk factors in routine settings, as they provide estimators such as positive and negative predictive values and help identify individuals at high risk. The inclusion of these individual risk factors in a more complex model, by means of multivariable regression, improved their predictive capacity. Since these factors do not present collinearity, each risk factor explains different aspects (variance) within the cohort, resulting in a better representation of ICU- and non-ICU-based COVID-19 patients. Ultimately, our multivariate designed model that included HLA, Horowitz PRS proxies and clinical information displayed an AUC value of 0.79. These results suggest that combined risk scores could be employed as a basis for improved care and treatment of COVID-19 patients.

## 5. Conclusions

GWAS analysis was performed via genotyping of >820 K genomic variants in a cohort of intensive care unit (ICU)-hospitalized patients and non-ICU-hospitalized COVID-19 patients. We identified four variants associated with COVID-19 severity with genome-wide significance (ICU hospitalization), and we designed a multivariate predictive model including HLA, PRS and clinical data which displayed an area under the curve (ACU) value of 0.79. Combination of human genetic information (i.e., HLA and PRS) with clinical data in our cohort was instrumental for the development of a model to assess the risk for COVID-19 severity.

## Figures and Tables

**Table 1:** Characteristics of ICU- and non-ICU-hospitalized patients with COVID-19.

Characteristics	All	Non-ICU	ICU	*p*-Value	n
Gender (n, %)				0.041	1104
Male	706 (63.9%)	583 (82.6%)	123 (17.4%)	---	---
Female	398 (36.1%)	348 (87.4%)	50 (12.6%)	---	---
Age (median, IQR)	62.0 [52.0;72.0]	62.0 [52.0;72.0]	63.0 [52.0;71.0]	0.507	1104
Weight (median, IQR)	81.0 [70.5;91.5]	80.0 [70.0;90.0]	84.0 [75.0;95.0]	0.004	663
BMI (median, IQ)	29.4 [26.1;32.4]	29.1 [26.0;32.2]	29.9 [27.0;32.9]	0.075	461
Oxygen therapy					
IMV (n, %)	117 (10.6%)	2 (1.71%)	115 (98.3%)	<0.001	1102
NIMV (n, %)	334 (32.8%)	192 (57.5%)	142 (42.5%)	<0.001	1018
Oxygen glasses (n, %)	899 (81.4%)	737 (82.0%)	162 (18.0%)	<0.001	1104
Venturi mask	378 (34.2%)	262 (69.3%)	116 (30.7%)	<0.001	1104
Comorbidities					
HTA (n, %)	465 (42.2%)	383 (82.4%)	82 (17.6%)	0.151	1103
DM (n, %)	209 (18.9%)	163 (78.0%)	46 (22.0%)	0.007	1103
Chronic cardiomyopathy (n, %)	108 (9.79%)	91 (84.3%)	17 (15.7%)	1.000	1103
Cardiac arrhythmia (n, %)	94 (8.52%)	78 (83.0%)	16 (17.0%)	0.822	1103
Valvulopathy (n, %)	40 (3.63%)	37 (92.5%)	3 (7.50%)	0.219	1103
Cardiac ischemia (n, %)	90 (8.16%)	72 (80.0%)	18 (20.0%)	0.306	1103
DM treatment (n, %)				0.093	978
No treatment	812 (83.0%)	715 (88.1%)	97 (11.9%)	---	---
Insulin	21 (2.15%)	20 (95.2%)	1 (4.76%)	---	---
Insulin + oral antidiabetics	21 (2.15%)	18 (85.7%)	3 (14.3%)	---	---
Oral antidiabetics	124 (12.7%)	100 (80.6%)	24 (19.4%)	---	---

ICU, intensive care unit; IMV, invasive mechanical ventilation; NIMV, non-invasive mechanical ventilation; HTA, hypertension; DM, diabetes mellitus.

**Table 2:** GWAS variants showing genome-wide association with COVID-19 severity.

Chr:pos(b38)	rsID	EA	OA	OR	ICU Frequency	Non-ICU Frequency	*p*-Value	Nearest Gene
17:74332949	rs58027632	T	C	1.26	0.106	0.031	3.19 × 10^−9^	KIF19
10:122498480	rs736962	G	A	1.49	0.053	0.007	3.04 × 10^−9^	HTRA1
10:122521643	rs77927946	A	C	1.50	0.049	0.007	5.98 × 10^−9^	DMBT1
X:39639346	rs115020813	T	G	1.34	0.064	0.006	7.23 × 10^−9^	LINC01283

Chr:pos(b38), chromosome and position on human genome build 38; rsID, lead variant rs number; EA, effect allele; OA, other allele; OR, odds ratio; ICU freq, variant allele frequency in ICU patients; non-ICU freq, variant allele frequency in non-ICU patients; nearest gene, the nearest or most plausible nearby gene.

**Table 3:** Loci with at least two markers showing a *p*-value < 10^−4^.

Cytogenetic Band	Gene	*p*-Value	Number of Variants with *p* < 10^−4^
1q43	LINC01139	<5 × 10^−5^	2
5q24.1	CYP1A2; CPLX3	<5 × 10^−5^	2
1q43	LOC105373220; MIR4426	<5 × 10^−5^	2
16q23.1	WWOX	<10^−5^	2
Xq21.33	MIR548	<5 × 10^−5^	2
6p22.1	HLA-G	<10^−4^	10
7p21.3	---	<10^−4^	2
7q34	CASP2; CLCN1	<10^−4^	2
8p23.1	CLDN23	<10^−4^	2

Cytogenetic band, chromosomal location of the locus; gene, associated gene(s) at the locus.

**Table 4:** HLA alleles significantly associated with risk (OR > 1) or protective (OR < 1) outcomes in ICU vs. non-ICU hospitalization of COVID-19 patients.

Allele	Adjusted *p*-Value	OR
A*01:01	0.002	0.431
DRB3*03:01	0.007	2.124
DPB1*10:01	0.022	2.193
B*45:01	0.031	3.422
C*02:02	0.036	0.432
DRB1*13:03	0.043	3.765
DQB1*05:02	0.043	0.128
DQB1*06:09	0.045	3.604
DRB4*01:01	0.049	1.446

Adjusted *p*-value; false discovery rate method adjusted *p*-value.

## Data Availability

The data presented in this study are available upon reasonable request from the corresponding author.

## Supplementary Materials

The following supporting information can be downloaded at: https://www.mdpi.com/article/10.3390/biom15030393/s1, Figure S1: Population stratification identified via multidimensional scaling (MSD) plot; Table S1: List of previously identified COVID-19 PRS SNPs and their proxies; Table S2: List of SNPs showing a significant association following comparison of hospitalized non-ICU- and ICU-based COVID-19 patients [25,26,27,43,51,52,53,54]; Table S3: Variants associated with COVID-19 severity located on locus 3p21.31 (codominant model); Table S4: Single-variable analysis based on PRS and phenotype data; Table S5: Multivariable analysis based on PRS, HLA and phenotype data.
