# Supplementary material for: Genetic Analysis and Predictive Modeling of COVID-19 Severity in a Hospital-Based Patient Cohort
Source: Biomolecules. 2025 Mar 10;15(3):393. doi: 10.3390/biom15030393 (PMC11940120; doi:10.3390/biom15030393)
Supplement: Supplementary file 1 [file biomolecules-15-00393-s001.zip › biomolecules-3407441-supplementary.pdf]

## Supplementary Files

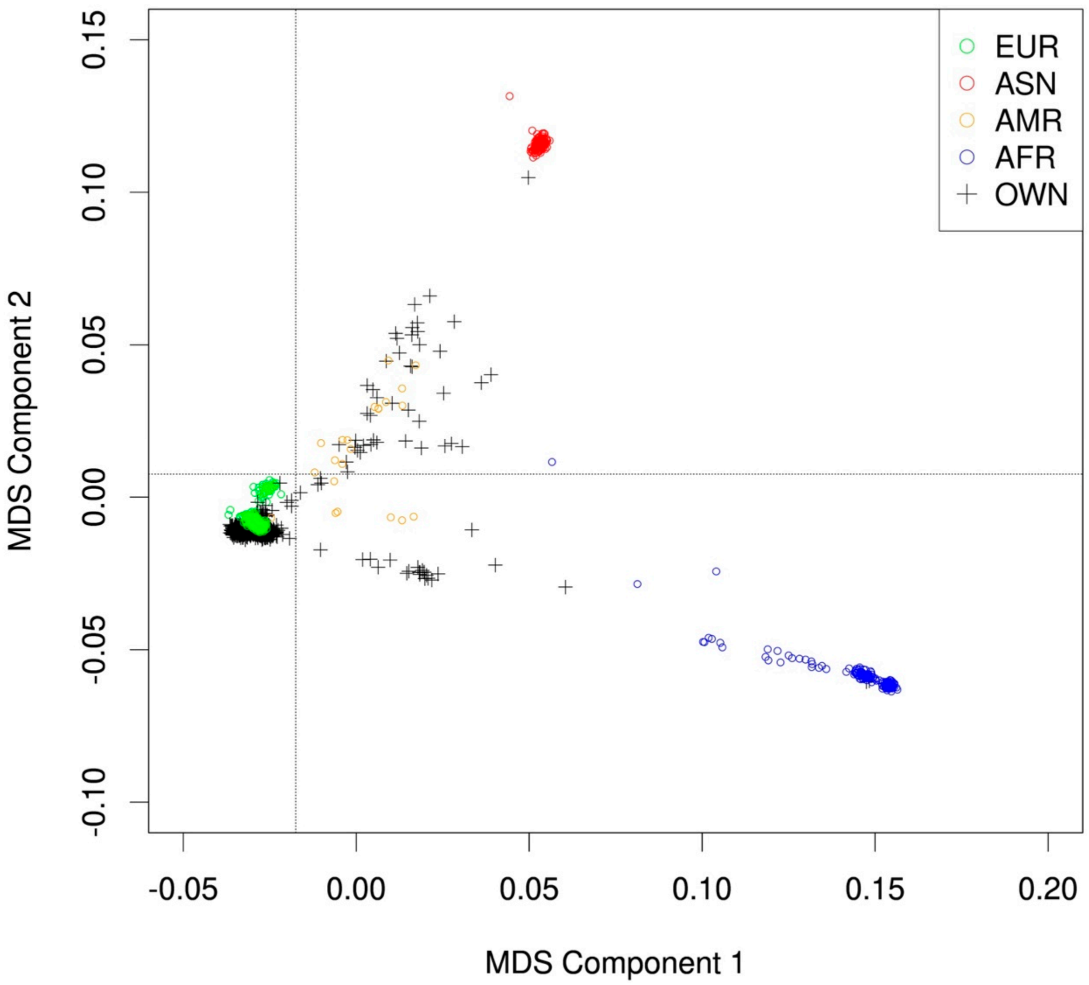


**Figure S1.** Multidimensional scaling (MDS) plot showing genetic clustering of study participants compared to reference populations from the 1000 Genomes Project. The x-axis and y-axis represent the first two MDS components, capturing the primary dimensions of genetic variation. Individuals are color-coded by population: European (EUR, green), Asian (ASN, orange), African (AFR, blue), and American (AMR, light orange), with study participants represented by black crosses (OWN).

**Table S1.** List of previously identified COVID-19 PRS SNPs [13] and their proxies. All proxies displaying LD $r^2$ values > 0.6 are listed.

| PRS SNPs | LD SNPs | $r^2$ value | OR | PRS weight |
|---|---|---|---|---|
| rs73064425 | rs13081482* | 1 | 2.14 | 0.7608 |
| | rs34288077 | 1 | - | - |
| | rs34326463 | 1 | - | - |
| | rs35044562 | 1 | - | - |
| | rs35081325 | 1 | - | - |
| | rs35731912 | 1 | - | - |

| | | | | |
|---|---|---|---|---|
| rs10735079 | rs6489882* | 0.99 | 1.29 | 0.2546 |
| rs4801778 | rs56104184* | 0.99 | 0.95 | 0.0512 |
| rs143334143 | rs72856718* | 0.99 | 1.85 | 0.6151 |
| rs11919389 | rs35117343* | 0.98 | 0.94 | 0.0618 |
| rs2236757 | rs2284549* | 0.97 | 1.28 | 0.2468 |
| rs2109069 | rs12610495* | 0.88 | 1.36 | 0.3074 |
| rs74956615 | rs34536443* | 0.82 | 1.59 | 0.4637 |
| rs9411378 | rs8176645* | 0.66 | 1.17 | 0.1570 |
| rs10070196 | rs1838700* | 0.60 | 1.05 | 0.0487 |
| rs1886814 | rs12660421 | 0.59 | - | - |
| rs2531743 | rs2531748 | 0.14 | - | - |

*, SNPs selected for PRS analysis; $r^2$ value, linkage disequilibrium parameter; PRS weight, individual polygenic risk score weight assigned to each SNP.

**Table S2.** List of SNPs showing a significant association following comparison of hospitalized non-ICU and ICU-based COVID-19 patients. The table includes SNPs reaching a significance threshold of $p \leqslant 10^{-4}$.

| Chr:pos(b38) | rsid | EA | OA | OR | SNP ICU freq | SNP non-ICU freq | p - value | Nearest gene | Previous observations |
|---|---|---|---|---|---|---|---|---|---|
| 17:74332949 | rs58027632 | T | C | 1.26 | 0.106 | 0.031 | $3.19x10^{-9}$ | KIF19 | - |
| 10:122498480 | rs736962 | G | A | 1.49 | 0.053 | 0.007 | $3.04x10^{-9}$ | HTRA1 | EUR[1]; POL[2,] |
| 10:122521643 | rs77927946 | A | C | 1.50 | 0.049 | 0.007 | $5.98x10^{-9}$ | DMBT1 | EUR[3] |
| 23:39639346 | rs115020813 | T | G | 1.34 | 0.064 | 0.006 | $7.23x10^{-9}$ | LINC01283 | - |
| 19:33639430 | rs2059876 | T | C | 1.10 | 0.40 | 0.26 | $1.44x10^{-07}$ | CHST8 | EUR[3] |
| 16:8970570 | rs11643976 | T | G | 1.22 | 0.12 | 0.04 | $4.00x10^{-07}$ | ENSG00000261392 | - |
| 16:78493701 | rs75464742 | A | G | 1.27 | 0.07 | 0.02 | $1.17x10^{-06}$ | WWOX | POL[2];EUR[3]; ALL[4] |
| 23:94356178 | rs62595441 | T | C | 1.16 | 0.11 | 0.03 | $1.86x10^{-06}$ | microRNA 548m | - |
| 16:78842560 | rs116965747 | G | A | 1.40 | 0.04 | 0.01 | $2.93x10^{-06}$ | WWOX | POL[2];EUR[3]; ALL[4] |
| 16:61584234 | rs17822636 | G | T | 1.09 | 0.32 | 0.20 | $3.81x10^{-06}$ | microRNA4426 | - |
| 22:23013975 | rs188925778 | T | G | 1.38 | 0.04 | 0.01 | $4.62x10^{-06}$ | RSPH14 | - |
| 13:96558588 | rs3848014 | C | T | 1.25 | 0.07 | 0.02 | $5.68x10^{-06}$ | HS6ST3 | EUR[3] |
| 20:756535 | rs3892648 | A | G | 1.19 | 0.10 | 0.04 | $8.47x10^{-06}$ | SCRT2 | ALL[4] |
| 9:20304998 | rs118097652 | A | C | 1.40 | 0.04 | 0.01 | $9.03x10^{-06}$ | MLLT3 | ALL[4] |
| 15:66681136 | rs2469155 | A | G | 0.92 | 0.13 | 0.27 | $1.09x10^{-05}$ | LINC01169 | - |
| 8:88797023 | rs11781588 | T | C | 1.07 | 0.59 | 0.44 | $1.11x10^{-05}$ | LOC105375630 | - |
| 6:29790218 | rs1611196 | C | T | 0.91 | 0.18 | 0.30 | $1.19x10^{-05}$ | HLA-G | SAU[5]; EUR[3,6,7] |
| 23:106162184 | rs140045164 | A | G | 1.21 | 0.06 | 0.01 | $1.30x10^{-05}$ | MUM1L1 | - |
| 7:98648166 | rs1905294 | C | T | 1.12 | 0.17 | 0.09 | $1.60x10^{-05}$ | NPTX2 | - |
| 16:49843026 | rs80335284 | A | G | 1.25 | 0.06 | 0.02 | $1.83x10^{-05}$ | ZNF423 | BL/AAM[7] |
| 23:79712267 | rs144924768 | T | C | 1.22 | 0.06 | 0.01 | $2.00x10^{-05}$ | ITM2A | - |
| 20:5253099 | rs6139672 | C | T | 1.28 | 0.05 | 0.01 | $2.04x10^{-05}$ | PROKR2 | - |
| 12:127652053 | rs67396890 | A | G | 1.07 | 0.53 | 0.39 | $2.16x10^{-05}$ | LOC105370068 | |
| 6:153102786 | rs514784 | C | G | 0.92 | 0.09 | 0.21 | $2.23x10^{-05}$ | RGS17 | EUR[4]; POL[2] |
| 1:238467020 | rs200659141 | T | G | 1.28 | 0.05 | 0.01 | $2.26x10^{-05}$ | LINC01139 | - |
| 1:238438513 | rs6667159 | C | T | 1.28 | 0.05 | 0.01 | $2.26x10^{-05}$ | LINC01139 | - |
| 16:61576315 | rs4784130 | T | G | 1.08 | 0.30 | 0.19 | $2.34x10^{-05}$ | LOC105373220 | - |
| 17:79875084 | rs57243117 | T | C | 1.37 | 0.04 | 0.01 | $2.45x10^{-05}$ | --- | - |

| | | | | | | | | | |
|---|---|---|---|---|---|---|---|---|---|
| 17:83204151 | rs34475269 | A | G | 0.93 | 0.30 | 0.44 | $2.46x10^{-05}$ | LOC101929650 | - |
| 8:8871787 | rs73524015 | A | G | 1.19 | 0.09 | 0.03 | $2.51x10^{-05}$ | MFHAS1 | EUR[3] |
| 10:91564468 | rs11817546 | A | G | 1.53 | 0.04 | 0.01 | $2.70x10^{-05}$ | PPP1R3C | - |
| 17:77620156 | rs28507999 | A | T | 1.10 | 0.22 | 0.11 | $2.80x10^{-05}$ | LOC100132174 | - |
| 23:99489341 | rs5921330 | G | A | 1.13 | 0.11 | 0.04 | $3.05x10^{-05}$ | --- | - |
| 2:120600105 | rs184890237 | T | C | 1.16 | 0.11 | 0.04 | $3.15x10^{-05}$ | --- | - |
| 15:74752123 | rs72547514 | A | G | 1.51 | 0.06 | 0.01 | $3.30x10^{-05}$ | CYP1A2 | EUR[3]; TUR[8] |
| 8:133579856 | rs72722104 | G | T | 1.10 | 0.23 | 0.14 | $3.33x10^{-05}$ | --- | - |
| 11:106587813 | rs12294363 | T | C | 1.30 | 0.08 | 0.02 | $3.36x10^{-05}$ | --- | - |
| 17:27363536 | rs55972629 | A | G | 1.22 | 0.06 | 0.02 | $3.46x10^{-05}$ | --- | - |
| 23:94366281 | rs1458878 | C | A | 1.05 | 0.56 | 0.38 | $3.47x10^{-05}$ | --- | - |
| 12:131361692 | rs9651789 | A | C | 0.93 | 0.17 | 0.30 | $3.60x10^{-05}$ | --- | - |
| 21:39981076 | rs2837342 | G | A | 1.07 | 0.44 | 0.31 | $3.64x10^{-05}$ | PCP4 | - |
| 13:58736161 | rs7987098 | G | A | 1.09 | 0.28 | 0.17 | $3.86x10^{-05}$ | --- | - |
| 19:57974824 | rs111645241 | A | G | 1.35 | 0.07 | 0.02 | $4.32x10^{-05}$ | C19orf18 | - |
| 1:25841295 | rs213633 | A | G | 1.08 | 0.33 | 0.21 | $4.41x10^{-05}$ | AUNIP | - |
| 7:10112903 | rs6463973 | T | G | 1.34 | 0.04 | 0.01 | $4.42x10^{-05}$ | --- | - |
| 2:124892929 | rs2791636 | T | C | 1.10 | 0.18 | 0.10 | $4.43x10^{-05}$ | CNTNAP5 | EUR[3]; ALL[4] |
| 6:29797514 | rs1629068 | G | A | 0.92 | 0.17 | 0.29 | $4.61x10^{-05}$ | HLA-G | SAU[5]; EUR[3,6,7] |
| 10:118672356 | rs138296613 | A | G | 1.24 | 0.06 | 0.02 | $4.79x10^{-05}$ | PRLHR | - |
| 4:185415311 | rs2289722 | C | T | 1.17 | 0.09 | 0.04 | $4.84x10^{-05}$ | UFSP2 | EUR[3] |
| 15:94570010 | rs7181323 | T | C | 1.07 | 0.60 | 0.46 | $4.88x10^{-05}$ | MCTP2 | EUR[3] |
| 1:29489133 | rs4078312 | T | C | 1.15 | 0.11 | 0.05 | $4.91x10^{-05}$ | LOC101928460 | - |
| 16:84237552 | rs3803640 | A | C | 1.10 | 0.21 | 0.12 | $5.08x10^{-05}$ | KCNG4 | - |
| 18:54506356 | rs8083639 | T | C | 1.26 | 0.05 | 0.02 | $5.09x10^{-05}$ | C18orf54 | - |
| 23:96543630 | rs138455513 | T | C | 1.14 | 0.10 | 0.03 | $5.72x10^{-05}$ | DIAPH2 | - |
| 15:74830184 | rs140289965 | A | C | 1.37 | 0.06 | 0.01 | $5.96x10^{-05}$ | CPLX3 | - |
| 9:82482479 | rs62576845 | T | G | 1.26 | 0.05 | 0.01 | $6.27x10^{-05}$ | LOC105376111 | - |
| 7:143328892 | rs4725613 | T | C | 1.07 | 0.52 | 0.40 | $6.76x10^{-05}$ | CLCN1 | - |
| 2:49896319 | rs116606423 | G | T | 1.23 | 0.06 | 0.02 | $6.81x10^{-05}$ | --- | - |
| 19:55479236 | rs73057171 | A | G | 1.28 | 0.04 | 0.01 | $6.85x10^{-05}$ | ZNF628 | - |
| 8:6058474 | rs55916457 | A | G | 1.20 | 0.08 | 0.03 | $7.02x10^{-05}$ | --- | - |
| 7:10135250 | rs73268663 | G | A | 1.36 | 0.03 | 0.01 | $7.32x10^{-05}$ | --- | - |
| 6:93095414 | rs13209950 | G | A | 1.09 | 0.24 | 0.14 | $7.32x10^{-05}$ | --- | - |
| 8:85962041 | rs139146754 | T | G | 1.27 | 0.05 | 0.01 | $7.45x10^{-05}$ | --- | - |
| 8:27245507 | rs17366947 | G | A | 0.94 | 0.29 | 0.42 | $7.50x10^{-05}$ | STMN4 | EUR[3] |
| 16:7749350 | rs2907356 | T | C | 1.22 | 0.06 | 0,.12 | $7.60x10^{-05}$ | --- | - |
| 14:81446174 | rs78345569 | A | C | 1.16 | 0.10 | 0.04 | $7.63x10^{-05}$ | LOC100506700 | - |
| 18:48033694 | rs56278643 | T | C | 1.10 | 0.18 | 0.10 | $7.66x10^{-05}$ | ZBTB7C | EUR[3];AFR[4] |
| 7:48279214 | rs2222648 | T | C | 1.07 | 0.35 | 0.24 | $7.98x10^{-05}$ | ABCA13 | - |
| 6:29806222 | rs1736976 | G | A | 0.92 | 0.23 | 0.35 | $8.01x10^{-05}$ | HLA-G | SAU[5]; EUR[3,6,7] |
| 6:29775786 | rs1633034 | G | A | 0.92 | 0.21 | 0.33 | $8.12x10^{-05}$ | HLA-G | SAU[5]; EUR[3,6,7] |
| 13:108766309 | rs9559407 | C | T | 1.21 | 0.06 | 0.02 | $8.13x10^{-05}$ | MYO16 | EUR[3] |
| 9:106795835 | rs75223861 | C | G | 1.15 | 0.09 | 0.04 | $8.14x10^{-05}$ | --- | - |
| 20:59496030 | rs76805868 | G | A | 1.28 | 0.05 | 0.01 | $8.34x10^{-05}$ | --- | - |
| 4:153273813 | rs4696448 | C | T | 1.07 | 0.43 | 0.31 | $8.40x10^{-05}$ | TRIM2 | EUR[3] |
| 6:29815287 | rs1077433 | A | G | 0.92 | 0.17 | 0.29 | $8.42x10^{-05}$ | HLA-G | SAU[5]; EUR[3,6,7] |
| 6:29804772 | rs1632987 | G | C | 0.92 | 0.17 | 0.29 | $8.42x10^{-05}$ | HLA-G | SAU[5]; EUR[3,6,7] |

| | | | | | | | | | |
|---|---|---|---|---|---|---|---|---|---|
| 6:29804618 | rs1632988 | A | G | 0.92 | 0.17 | 0.29 | $8.42\times10^{-05}$ | HLA-G | SAU[5]; EUR[3,6,7] |
| 6:29814693 | rs1736959 | T | C | 0.92 | 0.17 | 0.29 | $8.42\times10^{-05}$ | HLA-G | SAU[5]; EUR[3,6,7] |
| 6:29810206 | rs1736963 | A | G | 0.92 | 0.17 | 0.29 | $8.42\times10^{-05}$ | HLA-G | SAU[5]; EUR[3,6,7] |
| 21:41368561 | rs78736162 | G | A | 1.19 | 0.07 | 0.02 | $8.65\times10^{-05}$ | MX2 | EUR[3] |
| 20:56257537 | rs59328089 | A | G | 1.25 | 0.05 | 0.01 | $8.92\times10^{-05}$ | MC3R | - |
| 8:8708265 | rs10503394 | G | A | 1.08 | 0.32 | 0.21 | $8.96\times10^{-05}$ | CLDN23 | - |
| 1:183403540 | rs74914001 | C | T | 1.23 | 0.06 | 0.02 | $9.05\times10^{-05}$ | NMNAT2 | - |
| 6:29805732 | rs1632983 | T | C | 0.92 | 0.22 | 0.34 | $9.15\times10^{-05}$ | HLA-G | SAU[5]; EUR[3,6,7] |
| 8:52976231 | rs2588085 | T | C | 0.93 | 0.23 | 0.35 | $9.18\times10^{-05}$ | NPBWR1 | - |
| 8:8714870 | rs873064 | A | G | 1.08 | 0.32 | 0.21 | $9.21\times10^{-05}$ | CLDN23 | - |
| 16:82264843 | rs2873562 | A | G | 1.07 | 0.41 | 0.28 | $9.28\times10^{-05}$ | --- | - |
| 7:143301509 | rs3212147 | G | T | 1.22 | 0.06 | 0.02 | $9.59\times10^{-05}$ | CASP2 | - |
| 7:21696927 | rs73063727 | G | A | 0.90 | 0.05 | 0.14 | $9.60\times10^{-05}$ | DNAH11 | EUR[3] |
| 16:2902469 | rs138522704 | T | C | 1.60 | 0.06 | 0.01 | $9.82\times10^{-05}$ | FLYWCH1 | - |
| 2:15711224 | rs76517583 | A | G | 1.18 | 0.07 | 0.03 | $9.86\times10^{-05}$ | LOC101926966 | - |

Chr:pos(b38), chromosome and position on human genome build 38; rsid, lead variant rsid; EA, effect allele; OR, odds ratio; SNP ICU freq, frecuencie of the SNP in ICU patients;SNP non-ICU freq, frecuencie of the SNP in non-ICU patients; p-value, P value in variants; Nearest gene, the nearest or most plausible nearby gene; POL, Polish; EUR, European; ALL, European ancentry; AFR, African ancestry and South Asian ancestry; SAU, Saudi Arabian ancestry; BL, Black ancestry; AAM, African-American ancestry; TUR, Turquish ancestry.

References cited in this Table: [1], Chung J. et al. Genome-wide pleiotropy study identifies association of PDGFB with age-related macular degeneration and COVID-19 infection outcomes. *J. Clin. Med.* (2022) 12 (1), 109; [2], Słomian D. et al. Better safe than sorry-Whole-genome sequencing indicates that missense variants are significant in susceptibility to COVID-19. *PLoS One*. (2023) 18(1):e0279356; [3], Pairo-Castaineira E. et al. GWAS and meta-analysis identifies 49 genetic variants underlying critical COVID-19. *Nature*, (2023) 617 (7962) 764-768; [4], Thibord F. et al. A year of COVID-19 GWAS results from the GRASP portal reveals potential genetic risk factors. *HGG Adv.* (2022) 14;3(2):10009; [5], Alyami A. et al. Relationships Between Polymorphisms in HLA-G 3'UTR Region and COVID-19 Disease Severity. *Biochem Genet.* (2024) 1-22; [6], Bian S. et al. Genetic determinants of IgG antibody response to COVID-19 vaccination. *Am J Hum Genet.* (2024) 111(1):181-199; [7], Shelton J.F. et al. Trans-ancestry analysis reveals genetic and nongenetic associations with COVID-19 susceptibility and severity. *Nat Genet.* (2021) 53(6):801-808; [8], Bozkurt I. et al. Prognostic Value of CYP1A2 (rs2069514 and rs762551) Polymorphisms in COVID-19 Patients. *Balkan J Med Genet.* (2023) 26(1):35-42. References in bold indicate association $<10^{-8}$.

**Table S3**. Variants associated with COVID-19 severity located on locus 3p21.31 (codominant model).

| | | **CODOMINANT** | | |
|---|---|---|---|---|
| **Gene** | **SNP** | **Position** | **p-value** | **OR** |
| LZTFL1 | rs34288077 | 45847198 | 0.02 | A/A 1.00<br>A/G 1.73 |
| LZTFL1 | rs35624553 | 45825948 | 0.02 | A/A 1.00<br>A/G 1.58 |
| CCR3 | rs3091309 | 46261693 | 0.03 | G/G 1.00<br>A/G 0.87<br>A/A 3.09 |
| LARS2 | rs28793 | 45230833 | 0.03 | G/G 1.00<br>A/G 1.58<br>A/A 0.00 |
| LZTFL1 | rs35081325 | 45848429 | 0.03 | A/A 1.00 |

| | | | | |
|---|---|---|---|---|
| | | | | A/T 1.78 |
| | | | | C/C 1.00 |
| | | | | C/G 0.74 |
| LIMD1, LARS2 | rs267239 | 45594721 | 0.03 | G/G 1.36 |
| | | | | A/A 1.00 |
| | | | | A/C 1.50 |
| LARS2 | rs11130061 | 45424801 | 0.05 | C/C 2.04 |
| | | | | T/T 1.00 |
| | | | | T/C 0.66 |
| CCR3, CCR1 | rs3181077 | 46209161 | 0.05 | C/C 1.30 |
| | | | | G/G 1.00 |
| XCR1, CCR3, CCR1 | rs114721856 | 46063778 | 0.05 | A/G 0.00 |

**Table S4.** Single-variable analysis based on PRS and phenotype data.

| Variable | p - value | OR (95% CI) | AUC |
|---|---|---|---|
| PRS_Norm_Upper90 | 0.001 | 2.41 (1.43 - 3.9) | 0.550 |
| Sex | 0.030 | 0.64 (0.42 - 0.96) | 0.547 |
| Age | not sig | - | - |
| DIABETES | 0.005 | 1.80 (1.18 - 2.70) | 0.550 |
| GLASSES | 0.000 | 1.19 (1.78 - 6.86) | 0.567 |
| VENTIMASK | 0.000 | 1.71 (3.77 - 8.36) | 0.720 |

PRS_Norm_Upper90, poligenic risk score upper 90% threshold; OR (95% CI), odds ratio with 95% confidence intervale; AUC, area under the curve.

**Table S5.** Multivariable analysis based on PRS, HLA and phenotype data. PRS_Norm_Upper90 represents the polygenic risk score (PRS) norm upper 90 indicating that an individual's genetic predisposition to a specific disease is higher than at least 90% of the population. Significant principal components (PC2 and PC3), sex, clinical variables and HLA (HLA_pos, HLA alleles with OR > 1, HLA_neg: HLA alleles with OR <1) data are shown.

| Variable | OR (95% CI) | p - value | Significance |
|---|---|---|---|
| PRS_Norm_Upper90 | 2.20 (1.21 - 3.91) | 0.008 | ** |
| SEX | 0.82 (0.52 - 1.29) | 0.410 | |
| PC2 | 1.05 (1.01 - 1.10) | 0.009 | ** |
| PC3 | 0.95 (0.91 - 0.98) | 0.011 | * |
| DIABETES | 1.60 (0.99 - 2.55) | 0.049 | * |
| GLASSES | 2.33 (1.09 - 5.59) | 0.040 | * |
| VENTIMASK | 4.81 (3.13 - 7.54) | 0.000 | *** |
| HLA_Pos | 1.90 (1.46 - 2.47) | 0.000 | *** |
| HLA_Neg | 0.51 (0.34 - 0.71) | 0.001 | *** |

OR (95% CI), odds ratio with 95% confidence intervale; p - value; ***, p - value ≤ 0.001, ** p - value ≤ 0.005; *, p - value ≤ 0.05
